# Facial Neuralgia—Its Operative Treatment

**Published:** 1887-06

**Authors:** 


### *FACIAL NEURALGIA—ITS OPERATIVE TREATMENT.*

Dr. George R. Fowler (*Annals of Surgery*) sums up the experience of surgeons on the operative treatment of facial neuralgia thus:

1. Neuralgias of the fifth cranial nerve, of peripheral origin, which have resisted methods of treatment other than operative, may be expected to yield to the operation of neurectomy of the trunk or trunks whose branches are distributed by the painful area. In this class of cases, the neurectomy should be carried, if possible, to the point at which the nerve makes its exit from the cranium.

2. Cases of central origin should be first submitted to a limited neurectomy, conjoined with nerve-stretching, in the hope that the process of degeneration thus set up, together with the rest gained by interrupting the centripetally-conducted stimuli, may favorably influence the diseased central organ. In case of relapse, this may be repeated, provided the period of rest thereby gained corresponds to the length of time which Waller's investigations show to be usually occupied by the process of degeneration and regeneration. If no relief is gained, a similar operation should be performed upon all of the divisions of the fifth nerve. This failing, a complete neurectomy of each division accessible should be done; and, finally, ligature of the common carotid may be tried as a last resort.

3. In case of doubtful origin, a complete neurectomy followed, in cases which relapse, by ligature of the external and common carotid, in turn, hold out the best prospect of cure.

4. A complete neurectomy of the second division of the fifth necessarily involves the extirpation or destruction of the spheno-palatine ganglion; and to this fact, rather than to any intrinsic tendency of the ganglion itself to keep up the irritation causing the neuralgia, is to be attributed, in all probability, any increasing immunity from relapse claimed to have been obtained in those cases in which Carnochan's operation has been performed.

No patient should be denied, other things being equal, the chance which any one or all these operations in turn may give him of escap_ ing, even for a short time, the intolerable suffering incident to an intractable or otherwise irremediable facial neuralgia.

